# Cardiomyopathy in Celiac Disease: A Systematic Review

**DOI:** 10.3390/jcm13041045

**Published:** 2024-02-12

**Authors:** Stefan Milutinovic, Predrag Jancic, Adam Adam, Milan Radovanovic, Charles W. Nordstrom, Marshall Ward, Marija Petrovic, Dorde Jevtic, Maja Delibasic, Magdalena Kotseva, Milan Nikolajevic, Igor Dumic

**Affiliations:** 1 Internal Medicine Residency Program at Lee Health, Florida State University College of Medicine, Tallahassee, FL 32301, USA; smilutinovic@fsu.edu (S.M.); mdelibasic@fsu.edu (M.D.); 2 School of Medicine, University of Belgrade, 11000 Belgrade, Serbia; prjancic@gmail.com (P.J.); milannikolajevic10@gmail.com (M.N.); 3 Cardiology Fellowship Program, Cook County, Chicago, IL 60612, USA; adam.adam@cookcountyhealth.org; 4 Section of Hospital Medicine, Dartmouth-Hitchcock Medical Center, Lebanon, NH 03766, USA; radovanovic.milan@mayo.edu (M.R.); marshall.ward@hitchcock.org (M.W.); 5 Mayo Clinic College of Medicine and Science, Rochester, MN 55905, USA; nordstrom.cw@mayo.edu; 6 Department of Hospital Medicine, Mayo Clinic Health System, Eau Claire, WI 54703, USA; 7 Cardiology Fellowship Program, Icahn School of Medicine at Mount Sinai, New York, NY 10029, USA; marija.petrovic@mountsinai.org; 8 Elmhurst Hospital, New York, NY 11373, USA; jevticd@nychhc.org; 9 Francisian Health, Olympia Fields, IL 60461, USA; magdalena.kotseva@franciscanalliance.org

**Keywords:** cardiomyopathy, celiac disease, gluten-sensitive enteropathy, extraintestinal manifestations of celiac disease, Lane–Hamilton syndrome, idiopathic pulmonary hemosiderosis, anemia

## Abstract

(1) **Background**: Cardiomyopathy in celiac disease or celiac cardiomyopathy (CCM) is a serious and potentially life-threatening disease that can occur in both adults and children. However, data supporting the causal relationship between celiac disease (CD) and cardiomyopathy (CMP) are still inconsistent. The aim of this study was to review and synthesize data from the literature on this topic and potentially reveal a more evidence-based causal relationship. (2) **Methods**: The Preferred Reporting Items for Systematic Reviews and Meta-Analyses (PRISMA) guidelines were used to search Medline, Embase, and Scopus databases from database inception until September 2023. A total of 1187 original articles were identified. (3) **Results**: We identified 28 CCM patients (19 adult and 9 pediatric) with a mean age of 27.4 ± 18.01 years. Adult patients with CCM were predominantly male (84.2%) while pediatric patients were predominantly female (75%). The most common comorbidities associated with CCM were anemia (75%) and pulmonary hemosiderosis (20%). In 35% of patients, CCM occurred before the diagnosis of CD, while in 48% of patients, CCM and CD were diagnosed at the same time. Diagnosis of CD preceded diagnosis of CCM in only 18% of patients. Diagnosis of CCM is often delayed with an average, from the onset of symptoms to diagnosis, of 16 months. All patients were treated with a gluten-free diet in addition to guideline-directed medical therapy. At 11-month follow-up, cardiovascular improvement was seen in 60.7% of patients. Pediatric mortality was 33.3%, while adult mortality was 5.3%. (4) **Conclusions**: Clinicians should be aware of the possible association between CD and CMP, and we recommend CD work-up in all patients with CMP who have concomitant anemia. While we identified only 28 cases in the literature, many cases might go unreported due to a lack of awareness regarding CCM. A high degree of clinical suspicion and a prompt diagnosis of CCM are essential to minimizing the risks of morbidity and mortality, as the combination of a gluten-free diet and guideline-directed medical therapy can improve clinical outcomes.

## 1. Introduction

Celiac disease (CD), also known as gluten-sensitive enteropathy (GSE), is a chronic, immune-mediated disease that develops in genetically predisposed individuals due to their sensitivity to cereal gluten [1,2,3]. The prevalence of the disease in the United States is about 1%, but there is a significant geographical variability worldwide. The highest incidences are seen in Scandinavia and Turkey with prevalences of up to 3% [3]. Patients with CD frequently exhibit extraintestinal manifestations that are sometimes more pronounced than the commonly described gastrointestinal symptoms of abdominal pain, diarrhea, dietary nutrient deficiency, and malabsorption [1,2,3,4]. Well-documented extraintestinal manifestations include dermatitis herpetiformis, iron deficiency anemia (IDA), celiac ataxia, celiac hepatitis, and neuropsychiatric, hematologic, and thrombotic disorders [1,2,3,4,5,6,7].

Cardiomyopathy (CMP) can be classified into primary and secondary. Primary CMP refers to a group of diseases that affect the heart muscle itself, with abnormalities manifesting both structurally and functionally. It is important to note that these abnormalities are distinct from issues related to valves, coronary arteries, congenital conditions, or hypertension [8,9,10]. However, in secondary CMP, the previously mentioned factors play key pathophysiological roles in the development of heart muscle changes. The leading cause of secondary CMP is ischemia in the setting of coronary artery disease, subsequent blood flow disruption, and a decreased oxygenation of cardiomyocytes [11].

Dilated CMP is a chronic, progressive disease of the heart muscle that presents with unilateral or bilateral ventricle dilation, decreased left ventricular ejection fraction (LVEF), and usually progresses to congestive heart failure (CHF) [12]. Primary dilated CMP is idiopathic, often with a genetic/familial predisposition, whereas the secondary form results from a direct injury to the myocardium with commonly described causes such as infection, alcohol use, hypertension, illicit drugs, and medications [12].

Cardiac manifestations of CD are rarely reported, and there is a paucity of evidence on this topic [13]. Among patients with dilated, non-ischemic CMP, there is a notable prevalence of those with CD [14,15,16]. Celiac cardiomyopathy (CCM) is a serious and potentially life-threatening disease, sometimes requiring heart transplantation [17], and can occur in adult and pediatric populations [18]. A potential connection between CD and CMP has been previously identified; however, the data supporting this association are inconsistent [15,19,20,21].

The aim of this systematic review was to review and synthesize data from the literature on this topic to further explore the previously identified potential connection between CD and CMP.

## 2. Materials and Methods

We performed a systematic literature review of all CCM cases utilizing the Preferred Reporting Items for Systematic Reviews and Meta-Analyses (PRISMA) guidelines and searching the Medline (via PubMed search engine), Embase, and Scopus databases. This study is registered with the ResearchRegistry, and the unique identifying number is 1783. All reported articles were analyzed from database inception until September 1st, 2023. A total of 1187 original articles were identified that mention the following *MeSH* terms: “celiac OR gluten-sensitive enteropathy” AND “cardiomyopathy OR myocarditis OR cardiac OR heart” NOT “celiac trunk”. We only included cases with biopsy-proven CD and where the diagnosis of CMP was established by identifying new ventricular systolic or diastolic dysfunctions via transthoracic echocardiography (TTE) or cardiac magnetic resonance imaging (CMR). Other findings supportive of diagnosis included an elevation in cardiac enzymes, with or without a new onset of arrhythmia. We excluded cases that did not have biopsy-proven CD (including those with positive serologic testing for CD) and where a CMP diagnosis was not certain, or where there was a plausible alternative diagnosis (ischemic work-up was not performed or incomplete, viral causes were not excluded, etc.). We also excluded cases of myocarditis without abnormalities in the systolic or diastolic heart function. Furthermore, duplicate articles, articles in a language other than English, abstracts without comprehensive case descriptions, and narrative reviews were all excluded.

**Figure 1:**
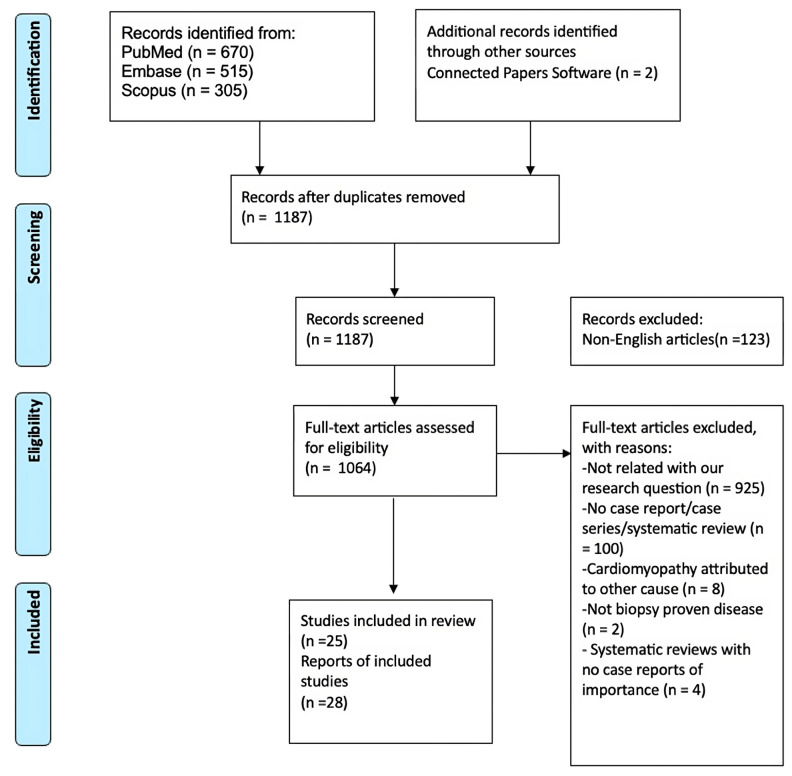
PRISMA flow chart.

Two authors (S.M. and P.J.) in blinded fashion independently identified and selected titles, abstracts, and full texts in the database search. Discrepancies in the selected articles were resolved by the senior author (I.D.). Additionally, reference lists of selected articles were searched to identify any additional cases for inclusion in accordance with previously established selection criteria. The flow chart of the detailed article selection and the final cases included in the analysis is illustrated in Figure 1.

For each case, we extracted patients’ demographic data, co-morbid conditions, presenting symptoms, physical exam findings, laboratory and imaging findings (including electrocardiograms [ECG], TTE, and other imaging techniques), treatment options, complications, and outcomes.

Data were analyzed through descriptive statistics and expressed as the mean ± standard deviation for continuous data, or as frequencies and percentages for categorical data. Percentages for categorical data were determined based on the total count of cases that provided specific categorical information. Not-reported values were excluded from calculations.

## 3. Results

### 3.1. Demographics and Comorbidities

Our systematic review identified 28 unique cases (19 adult and 9 pediatric patients) from 23 case reports and 2 case series, published from 1986 to 2022 [17,18,22,23,24,25,26,27,28,29,30,31,32,33,34,35,36,37,38,39,40,41,42,43,44]. The overall age range was from 3 to 70 years, with a mean of 27.4 ± 18.0 years. The pediatric mean age was 9.7 ± 4.8 years, and the adult mean age was 35.8 ± 15.2 years. The sex was reported in 27 cases (96.4%; not reported in one pediatric case). The adult patients were predominantly male (84.2%); however, the female sex was predominant among the pediatric population (75%). The reported comorbidities were anemia, lung hemosiderosis, diabetes mellitus type 1, stroke, hypothyroidism, depression, and atrial fibrillation (Table 1).

### 3.2. Presentation

Clinical presentation was reported in 25 cases (89.3%). Cardiovascular symptoms included dyspnea (*n* = 16), palpitations (*n* = 5), chest pain (*n* = 3), orthopnea (*n* = 3), paroxysmal nocturnal dyspnea (*n* = 3), and syncope (*n* = 2). Diarrhea (*n* = 9) was the most common gastrointestinal symptom, and constitutional symptoms (weakness/malaise/fatigue) were present in five patients. Almost all patients (95.5%) had at least one of the following: edema, crackles, heart murmur, or jugular vein distention (JVD). No patients presented with cardiogenic shock or developed cardiogenic shock during the disease course. Idiopathic pulmonary hemosiderosis (IPH), also known as Lane Hamilton syndrome, was present in four cases (20%).

**Figure 2:**
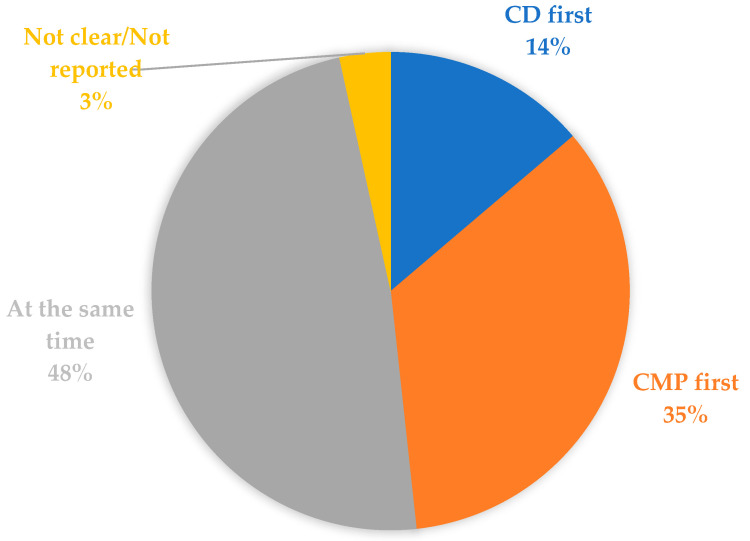
Diagnostic timeline. CD—Celiac Disease; CMP—Cardiomyopathy.

In five cases (17.8%), a diagnosis of CD had been established prior to the diagnosis of CMP. In 10 cases (35.7%), the diagnosis of CMP preceded the diagnosis of CD. For 12 patients, the diagnoses of CD and CMP were established simultaneously. The timeline was unclear in one adult case (Figure 2).

### 3.3. Evaluation

The time from symptom occurrence to final diagnosis was reported in 20 cases (71.4%). On average, it took 16.9 (range 0–84) months to diagnose a patient with CCM.

Laboratory results were often reported inconsistently. Troponin was reported in only five cases (17.9%), with four negative values and one mildly elevated value. The other laboratory values mentioned were hemoglobin (53.6%) and mean corpuscular volume (MCV) (25%) with median values at diagnosis being 7.7 g/dL (range 2.8–12.1) and 68.95 fL (range 49.7–74.0) respectively. Nearly all cases (*n* = 25, 89.2%) reported an elevation in at least one serologic marker of CD: anti-tissue transglutaminase antibody (IgA or IgG), endomysial antibody (IgA or IgG), anti-gliadin antibody (IgA or IgG), or deamidated gliadin antibody (IgA or IgG). Genetic testing was reported in one pediatric case and was positive for HLADQ B1*02, *03, and DRB1 *03*04 alleles [32]. No other genetic testing for possible underlying genetic CMP was reported in the case reports included in this study.

At the time of diagnosis, all cases (100%) were confirmed to have CMP via TTE, with 89.3% (*n* = 25) of cases having a quantified LVEF and the remaining three cases just reporting a diminished LVEF [27,42,43]. A total of 84.6% (*n* = 22) of cases reported dilated chambers on TTE. LVEF ranged from 10 to 50%. A reduced LVEF (EF < 50% or described as diminished) was present in 96.2% of the patients (*n* = 25). One patient had heart failure with preserved ejection fraction with an LVEF of 50%. The overall mean LVEF was 27.3 ± 11.5%. Elevated pulmonary artery pressure was reported in three cases, but none of the patients had confirmatory right catheterization for pulmonary hypertension. Pericardial effusion was reported in only two cases. Only three patients had a reported concurrent CD-associated autoimmune myocarditis diagnosis (two diagnosed via CMR imaging and biopsy, and one for which the method of diagnosis was not reported).

Only 25% (*n* = 7) of patients at admission had a New York Heart Association (NYHA) classification reported. Three patients were NYHA I, one patient was NYHA II, and three patients were NYHA III.

Sixteen patients (57.1%) had reported ECG abnormalities, including left bundle branch block (LBBB) (*n* = 10; 62.5%), right bundle branch block (RBBB) (*n* = 1; 6.3%), nonspecific intraventricular conduction delay (IVCD) (*n* = 1; 6.3%), and 2:1 AV block (*n* = 1; 6.3%). Additionally, four patients (25%) had tachyarrhythmias, and two patients (12.5%) had T-wave abnormalities (Table 2).

Additional diagnostic imaging performed included the following: CMR imaging in 4 patients (14.3%), coronary angiography (CA) in 11 patients (39.3%), coronary computed tomography angiography (CCTA) in 2 patients (7.1%), CT of the chest in 3 patients (10.7%), CT of the abdomen in 2 patients (7.1%), and 1 patient underwent an exercise stress test (3.6%). Endomyocardial biopsies were performed on five patients (17.9%).

Ischemic CMP was excluded in 13 adult cases (68.4%) with either anatomical or functional testing for coronary artery disease (CA, CCTA). Four cases with two relatively young adults (18- and 20-year-olds) did not report any ischemic work-up [17,28,33,41]. Also, one case had negative troponin on presentation and did not have an official ischemic CMP work-up, while one case underwent a negative stress test without additional testing for coronary artery disease [30,31].

### 3.4. Treatment, Complications, and Outcomes

LVEF improvement was reported in 13 (50%) out of the 26 patients (92.9%) who were started on a gluten-free diet (GFD) at the time of diagnosis. Proving causality between this change and a GFD is somewhat challenging partly because 75% (*n* = 21) of the patients received some form of additional guideline-directed medical therapy (GDMT) (Table 3 and Table 4). Additionally, only 50% of cases reported consistent GFD compliance. Patients reported improvement during their follow-up appointments, which averaged 11.4 months after GFD initiation. The earliest follow-up occurred at 3 weeks, while the latest was at 30 months. For patients compliant with their GFD, the average time until follow-up was 9.35 months (Table 4).

A total of nine cases (32.1%) reported the involvement of other organs with almost half of those (*n* = 4, 44.4%) involving the lungs. Of all the cases, nine described serious complications. The most frequent complication seen was ventricular tachycardia (*n* = 3, 30%) followed by other cardiac (e.g., heart block, atrial flutter) and non-cardiac (e.g., deep vein thrombosis) complications. Three intensive care unit (ICU) admissions were reported, including one pediatric patient [18,22,31].

During the last reported follow-up, 24 patients (85.7%) were alive. The pediatric mortality was 33.3% (three patients), with two dying in the hospital and one suddenly dying 2 years following diagnosis. The adult mortality was 5.3% (one patient) with the patient dying 1 month following admission with CMP diagnosis. The median follow-up time was 12.9 months, ranging from 1 to 60 months for cases that reported follow-up information [18,32,42,44].

## 4. Discussion

The literature on the association of these two entities is conflicting. Initially, a significant association was found by Curione et al., with a 5.8% prevalence of idiopathic CMP amongst patients with CD compared to 1.8% in the general population (*p* < 0.001) [15]. This was followed by two observational studies from Italy and Denmark, which also showed a possible association [45,46]. Additionally, De Bem et al. found increased endomysial antibodies in 2.6% of pretransplant patients suffering from idiopathic CMP [19]. It is important to acknowledge that each of these studies involved a relatively small patient sample (ranging from three to nine patients). More recent studies have also shown an association between these two diseases [47,48,49].

The largest population-based cohort study conducted so far included nearly 30,000 CD patients, among which 17 patients had CMP [21]. CD was found to have an associated risk for CMP development by 73%, with the highest risk within the first 5 years following CD diagnosis. The same study showed that a prior diagnosis of CMP was associated with a later diagnosis of CD. To the contrary, other studies have found no substantial associations between CD and CMP [16,20,50,51]. Elfström et al. did not find a statistical significance within adult (HR 1.7; 95% CI 0.4–6.5; *p* = 0.452) or pediatric (HR 0.8, 95% CI 0.2–3.7; *p* = 0.794) cohorts [51]. A Swedish retrospective cross-sectional study found LVEF to be better in patients with biopsy-proven CD (LVEF > 49%: 60.1% vs. 50.5%, *p* = 0.049) [50]. Thus far, there is no systematic literature review of case reports on this topic and our paper is the first to summarize data from case reports and case series regarding CD and CMP.

Aside from CMP, several different cardiac manifestations have also been attributed to the presence of CD [12,51,52,53]. Rhythm and conduction disturbances such as atrial fibrillation and AV block have all been described in the literature as possible complications of CD [52,53,54], some of which were seen in cases reported in this paper [28,41,55]. Moreover, vascular pathology, including accelerated atherosclerosis, thrombosis, and dysregulation of angiogenesis have all been linked to the presence of CD [13,56,57,58].

### 4.1. Pathophysiology

**Figure 3:**
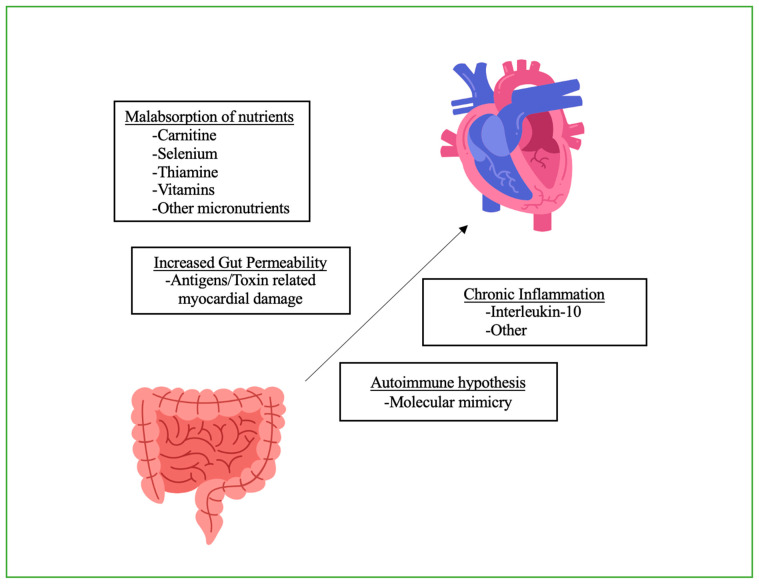
Celiac disease and its relationship with the heart.

The association between CD and CMP is complex and not fully understood. Several pathophysiologic mechanisms have been proposed, including the malabsorption of nutrients, a chronic inflammatory state, increased gut permeability, and autoimmune hypothesis (Figure 3) [33,35,40,59,60,61].

CD leads to the malabsorption of nutrients, which causes a range of complications such as anemia, coagulopathy, and thrombosis, all of which may lead to CMP, and CHF [62,63]. The progression of CHF has also been associated with nutritional deficiencies, making malabsorption a possible link between these two disorders [64]. Over half of CD patients have IDA, possibly due to malabsorption in the duodenum, which is the primary location of iron absorption [65,66], and a higher incidence of villous atrophy, which has been associated with a higher degree of IDA [67]. Carnitine deficiency has been known to occur in CD due to malabsorption. Carnitine is important for the oxidation of long-chain fatty acids, and its deficiency is proven to be associated with the development of CMP, likely due to long-term derangements of the cardiomyocyte energy metabolism [35,60,68,69]. Aside from this, the development of CMP may be exacerbated by the loss of selenium, thiamine, vitamins, and other micronutrients. CMP and CHF can lead to congestion and intestinal edema, which can further decrease the absorption of important nutrients [64].

The inflammatory theory asserts that chronic inflammation plays a central role in both CD and CCM [70]. Elevated levels of interleukin-4, interleukin-6, interleukin-10, and tumor necrosis factor-α, as well as other cytokines, were found in patients with CD [71]. Notably, increased levels of interleukin-10 are also found in patients with dilated CMP [72]. Additionally, myocarditis, as a common forerunner of dilated CMP has been linked with CD [59]. Hence, the inflammatory state in CD might explain both myocarditis and the development of CMP [73].

Intestinal permeability is increased in CD [54], which may permit the translocation of various antigens from the intestinal lumen, including toxins and infectious agents, which may damage the myocardium directly or indirectly through immune-mediated mechanisms [39]. Several studies indicate that molecular mimicry resulting in autoimmune injury might be a mechanism of cardiac damage, mirroring the way that the autoimmune response leads to intestinal damage. This mechanism was initially proposed by Chuaqui et al. with the immune disruption of actin cytoskeleton in myocardium [42]. Improvement in cardiac function, as well as in intestinal symptoms, following the introduction of a GFD would support this hypothesis [42,55,59]. Such mimicry can be seen between IgA antibodies produced in response to CD and the myocardium, further supporting an autoimmune nature of cardiac manifestations [74].

### 4.2. Clinical Presentation

CD has been associated with multiple autoimmune diseases including type 1 diabetes, autoimmune thyroid disease, selective IgA deficiency, rheumatoid arthritis, and connective tissue disorders [16,75,76,77,78,79]. Sometimes the symptoms and signs of these associated diseases are predominant, and awareness of their association with CD might help establish a diagnosis of asymptomatic or silent CD.

A pulmonary extraintestinal manifestation of CD is IPH, also known as Lane–Hamilton syndrome (LHS) [80]. Interestingly, IPH is also believed to be immunologically mediated, and a GFD is the recommended treatment [81]. Although described in the literature, IPH and CCM rarely occur simultaneously [30]. In this review, we found four cases of IPH and CCP occurring concomitantly [18,26,30,34]. Most of these cases presented with respiratory complaints or anemia and without gastrointestinal symptoms pertinent to CD. Since both CCM and IPH are very rare extraintestinal manifestations of CD, it might be that some people (due to still unrecognized genetic predisposition) might be more prone to develop these manifestations. In other words, the propensity in some individuals to develop rare extraintestinal manifestations might be associated with a higher likelihood of developing other rare manifestations. However, due to the limited number of cases and lack of basic science research on this topic, this currently remains only a hypothesis.

The presenting signs and symptoms of CCM usually coincide with those of CHF with the most common being dyspnea [82]. Most patients included in this review presented with signs of volume overload. Shock, being the most severe life-threatening complication, was not reported in the cases we included [82]. In this review, cardiac manifestations of CD were more common than gastrointestinal.

### 4.3. Diagnosis and Testing

When CD is suspected, serological testing for the presence of specific antibodies (anti-endomysial, anti-transglutaminase, etc.) is initially performed, followed by a small intestinal biopsy. However, if antibodies are absent but the clinical suspicion is strong, an intestinal biopsy can confirm the diagnosis of CD [66,83,84]. Nevertheless, esophagogastroduodenoscopy is not always safe and is not without risk of complications. In the case of acute decompensated CHF and severely reduced LVEF, biopsy is usually deferred until the patient is more clinically stable. In this review, nearly all cases reported an elevation in at least one serologic marker of CD, and all cases had biopsy-proven CD.

A CMP diagnosis is made based on imaging, primarily TTE [85]. Cardiac dysfunction in CD patients can be evaluated with newer techniques including two-dimensional speckle tracking echocardiography (2DSTE). This modality calculates regional and global myocardial deformation parameters such as strain and strain rate. Multiple studies have evaluated the usefulness of 2DSTE in the diagnosis of clinical or subclinical cardiac dysfunction in CD patients and have shown its superiority when compared to conventional TTE [86,87,88]. Cenk et al. found that strain and strain rate imaging is superior to conventional TTE for the evaluation of cardiac involvement in CD. The authors compared 20 CD patients and 20 healthy patients. There were no statistical differences in parameters obtained via conventional TTE between these two groups, including LVEF (67.8% and 68.5%). However, the strain values obtained from the LV in three out of eight segments were statistically higher in the control group (*p* < 0.05) [86]. Deveci et al. identified significant impairment of the LV radial and longitudinal strains in patients with CD compared with a healthy control group, while TTE showed no differences between patients with CD and the control group (including wall thickness, LV systolic, and diastolic parameters). Also, both intervention and control groups had normal LVEF [87]. Furthermore, El Amrousy et al. showed that the LV global longitudinal strain was significantly lower in children affected by CD when compared to healthy individuals [88]. While advanced echocardiography imaging techniques have demonstrated superior diagnostic capabilities for cardiac dysfunction, it is noteworthy that the cases included in our study did not utilize these imaging modalities. Instead, patients were diagnosed via conventional TTE. Additional modalities used in diagnosis in our cases were cardiac catheterization, CMR, and endomysial biopsy, mostly to rule out other causes of CMP.

Endomysial biopsy is the gold standard used to determine and confirm the cause of myocarditis, especially in patients with unexplained fulminant CMP, or unexplained new onset CHF of two weeks to three months duration associated with a dilated LV, new ventricular arrhythmias, AV blocks, or failure to respond to usual care within one to two weeks [89]. Biopsy can help in excluding other causes of CMP such as giant cell, lymphocytic, or sarcoid myocarditis. Once the diagnosis of CMP is established, biopsy or CMR can help determine the etiology, which may have therapeutic implications. In certain instances, patients might undergo serological and genetic testing for specific autoimmune and familial forms [85]. ECG and ambulatory Holter monitoring might be necessary in these patients if there is a concern for arrhythmogenic activity (e.g., in patients presenting with syncope) [85].

IDA in patients with CMP should arouse suspicion for CD [16,19]. De Bem et al. found 12.2% of cases with CMP to have CD and made a recommendation of screening these patients for CD [19]. In this review, 35.7% (*n* = 10) of patients had a diagnosis of CD made after a diagnosis of CMP was already established. The presence of IDA should prompt clinicians to have CD in differential diagnosis as one of the causes of non-ischemic CMP.

The main differential diagnoses that need to be excluded in patients with CCM are coronary artery disease, ischemic CMP, drug-induced, and CMP associated with infections. Most of the adult cases included in this review excluded ischemic CMP as a cause of LVEF worsening. Two adult cases reported relatively young patients (18 and 20 years old) that did not prompt ischemic work-up [17,28]. However, one of these cases had a CMR that did not show regional wall abnormalities, but rather signs of myocarditis [28]. In three cases, an ischemic workup was not mentioned, and the authors presumed CMP was related to CD-given improvement with a GFD [30,33,41].

### 4.4. Treatment

Currently, the only treatment proven to be effective in patients with CD is a GFD [90], but there are no specific recommendations regarding the treatment of CCM. Traditionally, CCM patients have been treated with a GFD in addition to adequate GDMT (Table 4).

As such, the assumption that adherence to a GFD leads to improvement in LVEF is hard to prove. There are conflicting data on whether a GFD is beneficial in patients with CCM. Some published data support that a strict GFD might have benefits on cardiac function [40]. Conversely, GFD-compliant patients in clinical remission have endoscopic abnormalities and histologic inflammation that persist for many years [70]. This could lead to an assumption that even with adherence to a GFD, patients may still have a residual risk for cardiac damage, particularly when considering the autoimmune hypothesis and molecular mimicry. Additionally, a GFD might play a more complex role in cardiovascular health regardless of CD, as GFD may lead to an increased consumption of fat and sugar in gluten-free dietary plans, further leading to the rise of long-term cardiovascular risk [91].

In this review, overall improvement was noted in most patients (*n* = 13; 50%). In total, 26 (92.9%) patients were started on a GFD, of which 22 (78.6%) were simultaneously treated with GDMT. With this therapeutic approach, showing a definitive causality between LVEF improvement and the GFD is somewhat challenging. Moreover, only half of the patients reported compliance with their GFD at follow-up visits. At an 18-month follow-up, Milisavljevic et al. reported a worsening in LVEF from 50% to 15–20% in a GFD non-compliant patient [31]. Interestingly, this patient was on a beta-blocker (BB) and angiotensin-converting-enzyme inhibitor (ACEi). Similar results without LVEF improvement were reported by Lodha et al. and Curione et al. [36,40]. In one case, after an initial LVEF improvement from 30% to 65%, the accidental ingestion of gluten led to a worsening of LVEF to 25% [41]. It is worth mentioning that four out of five cases that did not report being in GDMT in addition to on a GFD also showed significant improvement in LVEF [28,37,38,39] on follow-up TTE.

While a GFD is an effective treatment of gastrointestinal symptomatology in CD, clear and uniform evidence of reversing CMP through dietary intervention is lacking. Hence, our recommendation would be to follow the American College of Cardiology/American Heart Association heart failure guidelines and always treat CCM patients with adequate GDMT in addition to a strict GFD.

In recent years, other pathophysiology-driven strategies for treatments of CD have been developed [92]. The most promising agents are larazotide and latiglutenase, which work by stabilizing the enterocyte tight junctions and preventing mucosal degradation induced by gluten [93,94]. The inhibition of tissue transglutaminase-2 with ZED1227 showed safety and tolerability in a phase 1 clinical trial [95]. Additionally, immune modulation with anti-IL-15 antibody and pan-JAK inhibitor (tofacitinib) has been shown to have symptomatic benefits in CD patients [96,97]. Whether these drugs can be used in patients with CCM has yet to be investigated.

### 4.5. Complications and Outcomes

Considering the possible severity of CD presentation and duration of symptoms prior to diagnosis, many complications may arise in the course of this disease.

Rhythm abnormalities such as heart block and severe bradycardia requiring pacemaker implantation were reported by Anderson et al. and Milisavljevic et al., respectively [28,31]. Additionally, the case of Milisavljevic et al. was further complicated by an episode of atrial flutter treated with radiofrequency ablation [31]. Such changes in rhythm associated with CD are often described in the literature, with some cases showing significant improvement on a GFD [98].

In addition to the aforementioned cardiovascular complications, also reported were two cases of ventricular tachycardia [40,41], wide QRS treated with cardiac resynchronization therapy [26], and one case that resulted in a heart transplantation [17].

Out of four patients that died, three were pediatric cases. Due to the small sample size and the likelihood that these outcomes may have been random in nature, any correlation is difficult to substantiate amongst the cases presented in this paper.

## 5. Conclusions

In conclusion, patients with anemia and evidence of CMP should be evaluated for CD. Patients with CD may have a variety of cardiovascular complications, and clinicians should be aware of the possible association. With CCM, a high degree of clinical suspicion and prompt diagnosis are essential to minimize the risks of morbidity and mortality. Treatment with a GFD and implementing adequate GDMT are of paramount importance in treating patients with CCM. Regular follow-ups are needed to ensure compliance with dietary and medical therapy and to monitor for improvement in cardiac function. While we summarized all available case reports and case series in the last 35 years, additional rigorous prospective studies are needed to establish the association between CD and cardiac involvement and to elucidate the exact pathophysiology of this disease.

## 6. Limitations of Study

The limitations of our study are inherent to the nature of this type of literature review and include selection and publication bias. This systematic review included only 28 cases, which is admittedly a small sample. We included only articles published in the English language and in three databases, which put us at risk of missing some high-quality cases that did not meet our pre-selection criteria. In addition, all data were observational and due to small sample size statistics, descriptive.

## Figures and Tables

**Table 1:** Patients’ demographics, comorbidities, and clinical presentations.

Demographic Characteristics	*n*	Age Range (Years)	Mean Age ± SD (Years)	M:F Ratio
Adult	19 (67.9%)	18–70	35.8 ± 15.5	16:3
Pediatric	9 (31.1%)	3–17	9.7 ± 4.7	2:6 (1 NR)
Total	28 (100%)	3–70	27.4 ± 18.0	18:9 (1 NR)
**Comorbidities**				
Reported	20 (71.4%)			
Anemia	15 (75%)			
Idiopathic lung hemosiderosis	4 (20%)			
Atrial fibrillation, diabetes type 1, stroke, hypothyroidism, depression	Each 1 (5%)			
Not reported	8 (28.6%)			
**Clinical Presentation**				
Dyspnea	16 (64%)			
Diarrhea	9 (36%)			
Palpitations	5 (20%)			
Weakness/malaise, fatigue	5 (20%)			
Chest pain	3 (12%)			
Orthopnea	3 (12%)			
PND	3 (12%)			
Syncope	2 (8%)			
Not reported	3 (10.7%)			

Legend: M—Male; F—Female; SD—Standard Deviation; NR—Not Reported; VTE—Venous Thromboembolism; PND—Paroxysmal Nocturnal Dyspnea.

**Table 2:** Echocardiography, New York Heart Association classification, and electrocardiography findings.

Left Ventricular Ejection Fraction at Diagnosis	*n*	EF Range (%)	Mean EF ± SD (%)
Total reported	25 (89.3%)	10–50	27.3 ± 11.5
Adult	17	12–50	27.6 ± 11.3
Pediatric	8	10–45	27.6 ± 12.2
Not reported	3 (10.7%)	-	-
**New York Heart Association Classification**			
NYHA I	3		
NYHA II	1		
NYHA III	3		
NYHA IV	0		
Not reported	21 (75%)		
**Electrocardiography**			
Conduction abnormalities	13 (57.1%)		
LBBB	10 (62.5%)		
RBBB	1 (6.3%)		
IVCD	1 (6.3%)		
AV block	1 (6.3%)		
Tachyarrhythmias	4 (25%)		
T-wave abnormalities	2 (12.5%)		
Not reported or no abnormalities	12 (42.8%)		

Legend: EF—Ejection Fraction; SD—Standard Deviation; NYHA—New York Heart Association; LBBB—Left Bundle Branch Block; RBBB—Right Bundle Branch Block; IVCD—Intraventricular Conduction Delay; AV—atrioventricular.

**Table 3:** Published cases of celiac cardiomyopathy.

Reference/Year	Sex/Age	Timeline of Diagnosis	LVEF at Diagnosis	GFD	GDMT	Follow-Up LVEF
Mehra, 2022 [23]	M, 10	CMP	25–30%	Yes	Ivabradine	NR
Elnour, 2021 [24]	F, 33	Same time	15–20%	NR	BB, ARB, MRA, Ivabradine	NR
Meyer, 2021 [25]	F, 4	Same time	20%	Yes	NR	NR
Myrmel, 2021 [26]	M, 21	CMP	25%	Yes	BB, ARNi	35%
Bohra, 2020 [22]	F, 35	Same time	20%	Yes	“Inotrops”	55%
Patel, 2018 [27]	M, 19	CMP	“Severe systolic dysfunction”	Yes	Yes, but not specified	NR
Anderson, 2016 [28]	M, 20	CD	21%	Yes	NR	45% (1 y)
McGrath, 2016 [29]	M, 57	CMP	15%	Yes	BB, ACEi, MRA	63% (18 m) 70% (2 y)
Khilnani GC, 2015 [30]	M, 19	Same time	25%	Yes	BB	35% (2 y)
Poddar, 2014 [18]	M, 18	CMP	12%	Yes	BB, ACEi, MRA, Digoxin	25%
Poddar, 2014 [18]	F, 13	CD	10%	Yes, non-compliant	BB, ACEi, MRA, Digoxin	NR
Milisavljevic, 2012 [31]	M, 27	Same time	50%	Yes, non-compliant	BB, ACEi	20–25% (12 m) 15–20% (18 m)
Işikay, 2012 [44]	F, 13	Same time	32%	Yes	NR	29%
Boskovic, 2012 [32]	F, 3	Same time	39-45%	NR	NR	NR
Barrio, 2011 [17]	M, 24	Same time	24%	Yes	MRA, Digoxin	NR
Romagnoli, 2011 [33]	M, 66	Same time	25%	Yes	ACEi, Digoxin	NR
Dogan, 2010 [43]	F, 8	Same time	“Dilated Cardiomyopathy”	Yes	NR	NR
Narula, 2010 [34]	M, 13	Same time	26%	Yes	ACEi, Digoxin	NR
Uslu, 2010 [35]	F, 6	CD	46%	Yes	ACEi, Digoxin	“WNL”
Lodha, 2009 [36]	M, 48	CD	40–45% <25% *	Yes, non-compliant	BB, ACEi	“No improvement”
Glover, 2007 [37]	M, 36	Same time	15%	Yes	NR	25%
Gelfond, 2006 [38]	NR, 17	CD	15–20%	Yes	NR	36%
Goel, 2005 [39]	M, 70	CMP	45%	Yes	NR	65%
Curione, 2002 [40]	M, 40	CMP	38%	Yes	ACEi, Digoxin	42%
Curione, 2002 [40]	M, 32	CMP	25%	Yes	ACEi, Digoxin	30%
Curione, 2002 [40]	M, 26	CMP	36%	Yes, non-compliant	ACEi, Digoxin, BB added at follow-up	30%
Makhdoom, 2000 [41]	F, 49	CMP	30%	Yes	ACEi	65%, 25% **
Chuaqui, 1986 [42]	M, 34	Unclear	Diminished	Yes	NR	NR

* Two years after, on carvedilol, enalapril, and furosemide, but not on a GFD. ** After accidental gluten challenge. Legend: BB—Beta Blocker; ACEi—Angiotensin-Converting-Enzyme Inhibitors; ARB—Angiotensin Receptor Blocker, MRA—Mineralocorticoid Receptor Antagonist; WNL—Within Normal Limits; NR—Not Reported.

**Table 4:** Therapeutic approach.

Therapeutic Approach	*n* (%)
Gluten-free diet	26 (92.9%)
Not reported	2 (7.1%)
GDMT	20 (71.4%)
ACEi/ARB/ARNi	15 (75%)
Loop/thiazide diuretics	14 (70%)
Digoxin	9 (45%)
Beta-blocker	9 (45%)
MRA	5 (25%)
Other (dobutamine, ivabradine)	4 (20%)
GDMT started, but not specified	1 (3.6%)
SGLT-2i *	-
Not reported	7 (25%)

Legend: GDMT—Guideline-Directed Medical Therapy; ACEi—Angiotensin-Converting-Enzyme Inhibitors; ARB—Angiotensin Receptor Blocker; ARNi—Angiotensin Receptor/Neprilysin Inhibitor; MRA—Mineralocorticoid Receptor Antagonist; SGLT-2i—Sodium-Glucose Cotransporter 2 Inhibitors; * FDA approved in 2020 for Heart Failure Patients.

## Data Availability

All data are publicly available.
